# C-754-06. Multi-omic Alterations in Local Wound Environment of Pediatric Burn Patients

**DOI:** 10.1093/jbcr/irag033.034

**Published:** 2026-04-06

**Authors:** Julia A Penatzer, Ramya Chandran, Rajan K Thakkar

**Affiliations:** Burn Center at Nationwide Children's Hospital, Columbus, OH; Nationwide Children's Hospital, Columbus, OH; Nationwide Children's Hospital, Columbus, OH

## Abstract

**Introduction:**

The most common acute complications of pediatric burn injuries are infectious related while long-term complications include hypertrophic scarring and contracture formation. Prior studies have noted a heightened inflammatory state and immune dysfunction, systemically, but minimal data exists regarding the relationships between tissue-level responses and local outcomes (e.g., infection, graft failure), in pediatric burn injury. Our hypothesis is the local ‘omic tissue environment is altered following severe pediatric burn injury and exacerbated in those who go on to develop infection.

**Methods:**

Twenty-four pediatric burn patients with acute thermal injury who required clinically indicated excision/grafting procedures were enrolled, seven of which developed an infection (BI). Twenty-seven burn wound specimens and four unused, harvested skin graft tissues were obtained. Tissues were washed, dissected into two pieces, and snapped frozen or stored in RNAlater. RNA extraction was performed using RNeasy Plus Mini Kit and evaluated for quality and quantity. About 50-60 million paired end 150 bp sequence reads per sample were generated using the Illumina HiSeq 4000 platform. All fold change values were expressed as test condition/control condition. Transcripts were considered significantly differentially expressed using a 1% false discovery rate (DESeq2 adjusted *p*-value ≤0.01). For protein analysis, burn tissues were homogenized in cell lysis buffer, assayed for total protein concentration, and diluted to a total protein concentration of 200 μg/mL. Phosphoprotein and total protein were measured using commercial ELISA kits. A Kruskal-Wallis test with Dunn’s post-test was conducted to determine differences between groups. The differentially expressed genes (DEGs) and proteins were evaluated using QIAGEN's Ingenuity Pathway Analysis (IPA) to understand network and cellular pathway interactions.

**Results:**

Protein analysis indicated upregulation of pro-death signaling for BI compared to patients who did not (No BI). Thousands of DEGs were observed between No BI compared to BI groups; however, when comparing the burn patients who developed a systemic infection compared to those who developed a localized infection there were no DEGs. IPA analysis indicated the top canonical pathway for systemic infection to be related to cell cycle checkpoints, whereas the top pathway for burn wound infection was neutrophil degranulation.

**Conclusions:**

The results of our studies provide early evidence about the wound environment after burn injury that may lead or predict the development of adverse outcomes. Further we found there is little difference in the localized environment regardless of whether an infection occurs in the burn wound or systemically.

**Applicability of Research to Practice:**

This provides insight into developing therapeutics to target specific pathways to prevent infection and perhaps assist in wound healing.

**Funding for the study:**

NIH – NIGMS.

Wound Healing Foundation.

